# Pressure Support Ventilation (PSV) versus Neurally Adjusted Ventilatory Assist (NAVA) in difficult to wean pediatric ARDS patients: a physiologic crossover study

**DOI:** 10.1186/s12887-020-02227-1

**Published:** 2020-07-07

**Authors:** Giorgia Spinazzola, Roberta Costa, Daniele De Luca, Giovanna Chidini, Giuliano Ferrone, Marco Piastra, Giorgio Conti

**Affiliations:** 1 Department of Anesthesia and Intensive Care, Fondazione Policlinico Universitario Agostino Gemelli IRCCS, Largo F. Vito 1, 00168 Rome, Italy; 2 Division of Pediatric and Neonatal Critical Care, South Paris University Hospital, Medical Centers “A. Beclere” Assistance Publique-Hopitaux de Paris (APHP), Paris, France; 3 Pediatric Intensive Care Unit, Department of Anesthesia, Intensive Care and Emergency, Fondazione IRCCS Ca’ Granda, Ospedale Maggiore Policlinico, Milan, Italy; 4 Università Cattolica del Sacro Cuore, Roma, Italy

**Keywords:** Neurally adjusted ventilatory assist, Weaning, Pediatric acute respiratory distress syndrome, Patient-ventilator interaction, Mechanical ventilation

## Abstract

**Background:**

Neurally adjusted ventilatory assist (NAVA) is an innovative mode for assisted ventilation that improves patient-ventilator interaction in children. The aim of this study was to assess the effects of patient-ventilator interaction comparing NAVA with pressure support ventilation (PSV) in patients difficult to wean from mechanical ventilation after moderate pediatric acute respiratory distress syndrome (PARDS).

**Methods:**

In this physiological crossover study, 12 patients admitted in the Pediatric Intensive Care Unit (PICU) with moderate PARDS failing up to 3 spontaneous breathing trials in less than 7 days, were enrolled. Patients underwent three study conditions lasting 1 h each: PSV1, NAVA and PSV2.

**Results:**

The Asynchrony Index (AI) was significantly reduced during the NAVA trial compared to both the PSV1 and PSV2 trials (*p* = 0.001). During the NAVA trial, the inspiratory and expiratory trigger delays were significantly shorter compared to those obtained during PSV1 and PSV2 trials (Delay_trinsp_*p* < 0.001, Delay_trexp_*p* = 0.013). These results explain the significantly longer Time_sync_ observed during the NAVA trial (*p* < 0.001). In terms of gas exchanges, PaO_2_ value significantly improved in the NAVA trial with respect to the PSV trials (*p* < 0.02). The PaO_2_/FiO_2_ ratio showed a significant improvement during the NAVA trial compared to both the PSV1 and PSV2 trials (*p* = 0.004).

**Conclusions:**

In this specific PICU population, presenting difficulty in weaning after PARDS, NAVA was associated with a reduction of the AI and a significant improvement in oxygenation compared to PSV mode.

**Trial registration:**

ClinicalTrial.gov Identifier: NCT04360590 “Retrospectively registered”.

## Background

Partial ventilatory support modes are widely used both in the pediatric and adult population. However, patient-ventilator asynchrony represents, especially in the pediatric setting, still a problem. Pressure Support Ventilation (PSV) is also largely used both in the adult and in the pediatric population, even though studies have reported that cycling on-off algorithms, based on flow, may strongly affect patient-ventilator interaction [1, 2], causing several asynchronous events, such as prolongation or premature interruption of the mechanical insufflation, wasted effort or double cycling.

It is nowadays well known that asynchronous phenomena are frequent and are likely correlated to multiple factors, including machine characteristics and performance, as well as physiological factors affecting neural respiratory drive, muscle strength, as well as patient’s breathing patterns and respiratory mechanics. The latter two components are particularly crucial in infants and children, where the respiratory system characteristics and the fast breathing patterns may negatively interact with the flow-based ventilatory algorithms, particularly in difficult to wean patients who often show a high rate of asynchrony. Moreover, a worse patient-ventilator synchrony has been associated with increased days on mechanical ventilation and, consequently, increased risk to develop Ventilator Associating Pneumonia (VAP) and other infections [3–6].

These topics have particular relevance for the pediatric population affected by Pediatric Acute Respiratory Distress Syndrome (PARDS).

In a recent international observational study, Khemani and colleagues [7] reported the international PARDS incidence to be of 3.2% (95% CI 3.0, 3.4%) among Pediatric Intensive Care Unit (PICU) patients and 6.1% (95% CI 5.7, 6.5%) among patients on mechanical ventilation. Moreover, authors demonstrated that refractory hypoxemia is the main cause of death in 34% (41/121) of patients, multi-system organ failure in 43% (52/121), and brain death or neurologic injury in 28% (35/121) of patients.

Neurally Adjusted Ventilatory Assist (NAVA) is an innovative mode for assisted ventilation, which delivers proportioned positive pressure in response to the electrical activity of the diaphragm (Edi) [8]. Edi is not influenced by changes in muscle length, chest wall configuration, and/or lung volume [9–11], and correlates with phrenic nerve activity [12].

Several studies have demonstrated that NAVA improves patient-ventilator interaction in the pediatric population [13, 14]. Moreover, recent studies [15–17] have shown that, as observed for invasive mechanical ventilation, the application of non-invasive NAVA (NAVA-NIV) in children with Acute Respiratory Failure (ARF) is feasible reducing trigger delays and asynchronous events, improving patient-ventilator interaction compared to non-invasive pressure support (PSV-NIV).

The aim of this physiological single center, non-blinded, crossover study was to assess the effects of NAVA versus Pressure Support Ventilation (PSV) on patient-ventilator interaction in pediatric patients with difficulty in weaning from mechanical ventilation after moderate PARDS [18] of different origin.

## Methods

This study was performed in the Pediatric Intensive Care Unit (PICU) of the “Fondazione Policlinico Universitario A. Gemelli IRCCS” of Rome according to the ethical standard laid down in the 1964 Helsinki Declaration 16 [19]. The study was approved by the local institutional ethics committee (approval number A693/CE2010), and written informed consent was obtained from parents or legal guardians.

This study was recorded on ClinicalTrial register (registration number: NCT04360590 “Retrospectively registered”).

### Patient characteristics

From January 2011 to January 2014 all children in the age range of 1 month to 2 years that were admitted to the PICU of the “Fondazione Policlinico Universitario A. Gemelli IRCCS” of Rome, Italy were screened for the eligibility criteria of this investigation.

Inclusion criteria were a diagnosis of moderate PARDS [18], defined by the partial pressure of arterial oxygen to fraction of the inspired oxygen ratio (PaO_2_/FiO_2_) < 200 and an Oxygenation index (OI) > 8 and < 16.

Moreover, we included patients who presented one among the following criteria:

1. Respiratory Rate rate ≥ 2 SDs of the age-corrected range, the use of accessory muscles or paradoxical abdominal movement during respiration [20]
2. Need for a feeding tube and an indwelling arterial line, according to PICU routine care protocols.

Exclusion criteria were:

1. Hemodynamic instability requiring volume loading and/or positive inotropes
2. Severe respiratory impairment represented by hypoxemia (assessed as failure to maintain a PaO_2_ > 60 mmHg with fraction of inspired Oxygen [FiO_2_] = 0.6), by severe hypercapnia (PaCO_2_ > 55 mmHg), by acidosis (arterial pH [pHa] < 7.30), or recurrent apneas
3. Contraindication to nasogastric tube placement (such as recent facial trauma, esophageal varices, local malformations, esophageal or gastric surgery performed in the previous 6 months)
4. Increased intracranial pressure
5. Palliative care for end-stage oncologic disease
6. Neuromuscular, mitochondrial, metabolic, or chromosomal diseases presenting with neonatal hypotonia
7. Medullary lesions

### Study protocol

All children admitted to PICU for moderate PARDS, according to our PICU protocol, were evaluated for weaning verifying the following criteria: resolution or improvement of PARDS causes, hemodynamic stability, absence or progressive reduction of vasoactive drugs requirements, adequate level of consciousness (COMFORT≥18) [21], presence of spontaneous respiratory efforts, presence of the cough reflex, correction of significant metabolic and electrolyte imbalances and adequate gas exchange with Positive End Expiratory Pressure (PEEP) ≤8 cmH_2_O and FiO_2_ ≤ 0.5.

Patients who fulfilled these criteria underwent a spontaneous breathing trial (SBT) with Continuous Positive Airway Pressure (CPAP) of 5 cmH_2_O for 30 to 120 min.

All patients who failed up to 3 SBTs in less than 7 consecutive days [22], and presenting at least two of the following signs: diaphoresis, nasal flaring, tachycardia (Heart Rate ≥ 40 bpm), arrhythmias, hypotension, apnea, P_ET_CO2 increase > 10 mmHg, arterial pH decrease < 7.32, PaO2 < 60 mmHg with a FiO_2_ ≥ 0.40 (PaO_2_/FiO_2_ ≤ 150) [23], were enrolled in the study, as they were considered at high risk of asynchrony phenomena.

After enrollment, the standard nasogastric tube of each patient was replaced with a specific nasogastric tube (Edi catheter) with an array of eight bipolar electrodes mounted at its distal end (Getinge Critical Care, Solna, Sweden). The initial placement was directed by measuring the distance from the patient’s xiphisternum to the tragus of the ear and then extending the measurement until the nose. The Edi catheter was then inserted to the corresponding depth. Catheter positioning was carried out through a specific function of the Servo I ventilator (Getinge, Critical Care, Solna, Sweden), called Edi catheter positioning [24]. Confirmation of its appropriate placement was achieved viewing the online electrical displays from the catheter: the presence of a good quality Edi trace, with P waves displayed by the central electrodes, indicates optimal positioning, with the array spanning equally the diaphragm in both caudal and cranial directions [15–17, 25].

At enrollment, all patients were ventilated in PSV mode with a Servo-I ventilator set to obtain a Tidal Volume (Vt) of 6–7 ml/Kg, with a PEEP level targeted to obtain a peripheral oxygen saturation (SpO_2_) ≥92%. During PSV, the flow trigger sensitivity was adjusted at the lowest level to avoid auto-triggering phenomena, while the expiratory cycling-off was adjusted to obtain the best synchronization, according to flow/pressure tracings. To determine the corresponding NAVA level, able to achieve a similar peak inspiratory airway pressure to that obtained in PSV, a dedicated function called NAVA Preview was used.

All patients underwent three study conditions, lasting 1 h each: PSV1, NAVA, PSV2. The last 5 min of each trial were recorded with a specific software (Nava Tracker V 2.0 Maquet Critical care, Solna, Sweden) and data was stored for further analysis. The minutes continuously recorded during each trial were 5, then we analyzed all the breaths during the middle minute (i.e. the third minute).

### Measurements

The Airflow (V′), the Airway Pressure (Paw) and the Electrical Activity of the diaphragm (Edi) were obtained from the ventilator through a RS232 interface (sampling rate 100 Hz) and recorded by NAVA Tracker software. Data were further processed, filtered and analyzed by a specific software (NAVA Merger and ICU Lab 2.5, respectively, KleiStek, Advanced Electronic System, Rome, Italy).

On the flow (V′) tracing, we measured the mechanical respiratory rate (RR_mech_) and mechanical inspiratory and expiratory time (Ti_mech_ and Te_mech_), as well as the total breath duration (Ttot_mech_). By integrating the Flow on time, we estimated the Tidal volume (Vt) delivered from the ventilator to the patient. Also, we measured VT_mech_ (defined as the amount of volume delivered by the ventilator during the mechanical inspiratory phase and calculated as the volume generated between the opening of the inspiratory valve and the expiratory cycling off) and VT_neu_ (defined as the volume delivered during the neural inspiratory phase and calculated as the amount of volume generated from the onset of Edi swing to its peak).

By analysing the Edi tracing, we calculated the patient neural respiratory rate (RR_neu_) and the patient inspiratory and expiratory time (Ti_neu_ and Te_neu_). The former was calculated as the time between the onset of Edi swing and its peak, and the latter as the time between the Edi peak and the onset of the following Edi swing [24].

To estimate the asynchrony rate, we calculated the asynchrony index (AI), which is the ratio between the number of asynchronous events and the total respiratory rate, expressed as percentage [15]. An AI> 10% was considered a high rate of asynchrony. The major asynchrony events observed and analysed were Wasted Efforts (WE) (defined as a patient inspiratory effort not assisted by the ventilator), Auto-Trigger (AT) (defined as a mechanical insufflation in absence of a patient inspiratory effort) and Late Cycling (LC) (defined as a cycle with the mechanical inspiratory time greater than twice the patient’s neural time).

The inspiratory trigger delay (Delay_trinsp_) was calculated as the time lag between the onset of the Edi swing and the onset of ventilatory assistance, evaluated on Paw tracing. Similarly, the expiratory trigger delay (Delay_trexp_) was determined as the time lag between the Edi peak and the end of mechanical assistance measured on the Paw tracing.

To evaluate asynchrony, we measured the Vt_neu_/Vt_mech_, as the percentage of Vt delivered during the patient’s inspiratory phase, and the time of synchrony (Time_sync_) defined as the time during which the patient’s inspiratory effort and the ventilatory assistance are in phase. The time during which respiratory effort and ventilator assistance were synchronous, indexed to Ti_neu_ (Time_sync_/Ti_neu_) was also computed [26–28].

The amount of inspiratory effort was calculated as the Pressure Time Product of Edi per breath and per minute (PTPEdi/breath and PTPEdi/min) defined as the area under the Edi trace from the neural inspiration to the end of the neural expiration.

The neuroventilatory efficiency (Vt/Edi) was defined as Vt divided by the integral of the inspiratory Edi (∫Edi). The Edi time integral (mean Edi*Ti*RR) was calculated as an indicator of inspiratory electrical energy expenditure.

The neuro-ventilatory efficiency index (Vt × kg PBW/∫Edi) was calculated to compute the amount of tidal volume correlated to a specific patient inspiratory demand per breath [29].

In addition, at the end of each trial, the gas exchange values (pHa, PaO_2_, PaCO_2_, PaO_2_/FiO_2_ ratio) and hemodynamic variables (Heart Rate, Systolic Arterial Pressure, Diastolic Arterial Pressure, Mean Arterial Pressure) were registered.

### Endpoints

The primary endpoint of the study was the measurement of the AI during each study condition. The secondary endpoints were the variables describing patient-ventilator interaction (expressed as inspiratory and expiratory trigger delays, time of synchrony, Vt_neu_/Vt_mech_), PTPEdi/breaths and PTPEdi/min, neuro-ventilatory efficiency index, RRmech, RRneu, PeakPaw, PeakEdi and gas exchange values (pHa, PaO_2_, PaCO_2_, PaO_2_/FiO_2_ ratio) during the study.

### Statistical analysis

Given the physiological design of the study, we did not perform a formal sample size calculation. In consistency with previous investigations on this topic [13, 30], we enrolled 12 patients. Data distribution was assessed with the Kolmogorov-Smirnov test. Continuous variables with normal distributions were expressed as means and Standard Deviation and assessed with the Student t-test. Continuous variables with non-normal distributions were expressed as medians and interquartile ranges (IQR) and assessed with the Mann-Whitney test. Frequencies were compared with the chi-square or Fisher exact test, as appropriate. The analysis of variance (ANOVA) for repeated measures was performed to detect significant differences between the single experimental settings. *P* values < 0.05 were considered statistically significant. MedCalc Statistical Software version 14.12.0 (MedCalc Software bvba, Ostend, Belgium; http://www.medcalc.org; 2014) was used for statistical analysis.

## Results

### Baseline characteristics

**Fig. 1:**
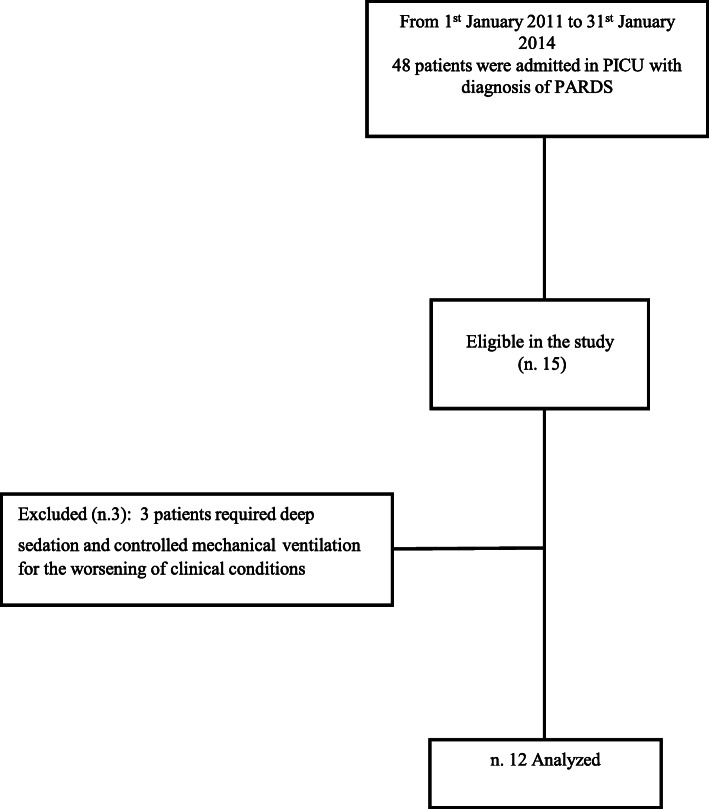
Study flow diagram

From January 1st 2011 to January 31st 2014, 48 pediatric patients were admitted in PICU with a diagnosis of moderate PARDS. Fifteen patients were eligible in the study after they failed 3 attempts of SBT; 3 patients were excluded due to worsening of the clinical conditions requiring deep sedation and controlled mechanical ventilation. The remaining 12 patients were enrolled in the study (Fig. 1).

The main clinical characteristics of our patients are shown in Table 1.

**Table 1:** Baseline patient characteristics

Patient	Age (Months)	Weight (Kg)	PARDS Cause	Comorbidities
**1**	**10**	**8**	**Pneumonia**	**Prematurity**
**2**	**1**	**3**	**Pneumonia**	**Onphalocele, Sepsis**
**3**	**2**	**4.5**	**V. Bronchiolitis**	**NONE**
**4**	**5**	**3.8**	**Pneumonia**	**NONE**
**5**	**2**	**4**	**V. Bronchiolitis**	**NONE**
**6**	**3**	**3**	**Pneumonia**	**Prematurity**
**7**	**2**	**4.5**	**V. Bronchiolitis**	**NONE**
**8**	**11**	**10**	**Pneumonia**	**Prematurity**
**9**	**6**	**12**	**Pneumonia**	**Pulmonary Hypertension, BPD, NEC**
**10**	**12**	**10**	**Pneumonia**	**Burn**
**11**	**24**	**6.7**	**Pneumonia**	**Prematurity**
**12**	**1**	**5**	**Pneumonia**	**NONE**
**Mean ± SD**	**6.58 ± 6.77**	**6.20 ± 3.07**		

*Abbreviations*: *SD* standard deviation, *V* viral, *NEC* necrotizing enterocolitis, *PARDS* pediatric Acute Respiratory Distress Syndrome, *Kg* kilogram

Ventilator settings did not differ for FiO_2_ (PSV1: 0.35 [0.34–0.42]; NAVA: 0.35 [0.34–0.42] and PSV2: 0.37 [0.34–0.45], respectively; p: 0.94) and PEEP level (PSV1: 6 [5–7] cmH_2_O; NAVA: 6 [5–7] cmH_2_O and PSV2: 6 [5–7] cmH_2_O, respectively; p:1). The Pressure Support level was set at 6 [5–9.5] cmH_2_O during PSV1 and PSV2 trials, while NAVA level was set at 1.45 [1.15–1.7] cmH_2_O/μV.

The Vt/Kg PBW values were similar in the three trials (PSV 1: 7.7 [6.6–10.52]; NAVA 9.23 [6.93–12.64] and PSV2: 6.4 [5.38–8.20] ml, respectively; *p* = 0.14). Also, no difference was observed in PeakPaw during each trial (PSV1 13.52 [11.29–21.82]; NAVA: 14.65 [12.32–28.20] and PSV2 17.50 [15–23.35] cm H_2_O, respectively; *p* = 0.47).

All enrolled patients completed each phase of the study, without interruptions. No major adverse events such as hemodynamic instability, bradycardia requiring chest compression, cardiac arrest, or severe hypercapnia were reported during the study.

### Primary endpoint

**Fig. 2:**
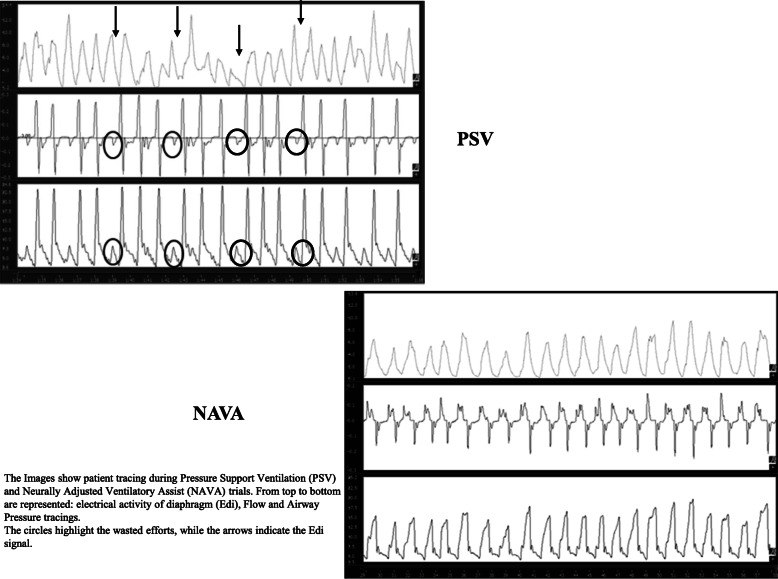
Patent tracings. The Images show patient tracing during Pressure Support Ventilation (PSV) and Neurally Adjusted Ventilatory Assist (NAVA) trials. From top to bottom are represented: electrical activity of diaphragm (Edi), Flow and Airway Pressure tracings. The circles highlight the wasted efforts, while the arrows indicate the Edi signal

The primary endpoint (AI) is shown in Table 2. AI was significantly reduced during the NAVA trial compared to both PSV1 and PSV2 trials (*p* = 0.001). Moreover, the number of wasted efforts and auto-triggering events was significantly higher during both PSV trials with respect to NAVA trial (AT *p* = 0.003, WE *p* < 0.0001) (Fig. 2). We did not observe significant differences in terms of late Cycling during the three trials (*p* = 0.176).

**Table 2:** Primary endpoints

	PSV1	NAVA	PSV2	***P***
**Asynchrony Index (%)**	13.6 [8–15.7]	1.7 [0–2.4]	10 [6.5–20]	**0.001**
**Auto Triggering (n/min)**	1.5 [0–3.5]	0 [0–0]	1 [0.2–3]	**0.003**
**Wasted efforts (n/min)**	1 [2–4]	0 [0–0]	2 [1–3.8]	**< 0.0001**
**Late Cycling (n/min)**	1.5 [0–4]	1 [0–1]	2 [1–5]	0.176

Data are expressed as median (interquartile range: 25th and 75th percentile)

*Abbreviations*: *N/min* number per minute, *%* percentage, *PSV* pressure support ventilation, *NAVA* neurally adjusted ventilatory assist

### Secondary endpoints

The secondary endpoints are shown in Table 3.

**Table 3:** Secondary endpoints

	PSV1	NAVA	PSV2	***P***
**Delay**_**Trinsp**_**(msec)**	116 [97–139.5]	27 [22–39]	125 [93–139]	**< 0.001**
**Delay**_**Trexp**_**(msec)**	91 [75–186]	45 [15–68]	82 [70–140]	**0.013**
**Time**_**sync**_**(sec)**	0.17 [0.13–0.21]	0.41 [0.37–0.51]	0.15 [0.13–0.18]	**< 0.001**
**Time**_**sync**_**/Ti**_**neu**_	0.60 [0.46–0.80]	0.96 [0.93–0.99]	0.67 [0.50–0.74]	**< 0.001**
**Neuroventilatory efficiency index (Vt/∫Edi) (mL × kg PBW/**μVs**-1)**	0.82 [0.33–2.68]	0.51 [0.30–1.60]	0.42 [0.21–0.98]	0.28
**Vt**_**neu**_**/Vt**_**mech**_**(%)**	100 [83–100]	100 [94–100]	100 [91–100]	0.60
**PTPEdi/min (μV/sec/min)**	1.21 [0.87–4.63]	1.79 [0.89–3.63]	2.09 [1.23–8.25]	0.48
**PTPEdi/breath (μV/sec)**	2.75 [2–8.35]	2.83 [2.1–8.04]	3.82 [1.97–13.16]	0.61
**PeakPaw (cmH**_**2**_**O)**	13.52 [11.29–21.82]	14.65 [12.32–28.20]	17.5 [15–23.35]	0.47
**PeakEdi (μVs)**	6.63 [4.62–21.51]	16.32 [7.52–25.46]	8.83 [5.19–33.10]	0.78
**Mean Arterial Pressure (mmHg)**	70 [65–84]	72 [66–78]	67 [63–70]	0.36
**HR (bpm)**	149 [140–154]	150 [130–154]	147 [137–155]	0.33
**PaO**_**2**_**/FiO**_**2**_**ratio**	232 [198–284]	288 [224–337]	228 [197–291]	**0.004**
**PaCO**_**2**_**(mmHg)**	34 [31–41]	32 [29–42]	32 [28–35]	0.62
**pHa**	7.44 [7.41–7.46]	7.43 [7.42–7.45]	7.45 [7.43–7.48]	0.50

Data are expressed as median (interquartile range: 25th and 75th percentile)

*Abbreviations*: *Delay*_*Trinsp*_ inspiratory trigger delay, *Delay*_*Trexp*_ expiratory trigger delay, *Time*_*syn****c***_ time of synchrony, *Edi* electrical activity of diaphragm, *sec* seconds, *Vt* tidal Volume, *∫Edi* Edi time integral, *Vt*_*neu*_ neural tidal volume, *Vt*_*mech*_ mechanical tidal volume, *msec* milliseconds, *Kg* Kilograms, *PBW* predicted body weight; ml: milliliters, *PTPEdi* pressure time product of Edi per breaths and per minute, *Paw* airway pressure, *μV* microvolt; min: minute, *%* percentage, *bpm* beats per minute, *HR* heart rate, *PaO*_*2*_*/FiO*_*2*_ ratio partial pressure of arterial oxygen to fraction of the inspired oxygen ratio, *PaCO*_*2*_ arterial partial pressure of carbon dioxide, *mmHg* millimetres of mercury, *pHa* arterial pH

During the NAVA trial, the inspiratory and expiratory trigger delays were significantly shorter compared to the delay values observed during PSV1 and PSV2 trials (Delay_trinsp_*p* < 0.001, Delay_trexp_*p* = 0.013): these results explain the significantly longer Time_sync_ observed during the NAVA trial (*p* < 0.001). Compared to both PSV trials, NAVA significantly improved Time_sync_/Ti_neu_ (*p* < 0.001). We did not observe significant differences in terms of Vt_neu_/Vt_mech_ during NAVA and PSV trials. During all trials, we did not observe significant differences in the amount of inspiratory effort evaluated through the analysis of PTPEdi/breath, PTPEdi/min and neuro-ventilatory coupling expressed by the neuro-ventilatory efficiency index (PSV1: 0.82 [0.33–2.68], NAVA: 0.51 [0.30–1.60], PSV2: 0.42 [0.21–0.98], respectively, *p* = 0.28).

Furthermore, we did not observe significant differences in terms of RRneu and RRmech between the trials, although there was a trend toward a rise of RRmech and RRneu moving from the NAVA trial to the second trial in PSV (RRmech PSV1: 54.5 [37–60.6], NAVA: 46.5 [41.5–62], PSV2: 55 [45.5–62.5], respectively; *p* = 0.82) (RRneu PSV1: 58 [37.5–62.5], NAVA: 46.5 [41.5–62], PSV2: 57.5 [45.5–63], respectively; *p* = 0.76). During all trials, no differences in terms of PeakPaw and PeakEdi were demonstrated.

In terms of gas exchanges, PaO_2_ significantly improved when NAVA trial was compared to both PSV trials (*p* = 0.025). PaO_2_/FiO_2_ ratio, significantly improved during NAVA trial, compared to both PSV1 and PSV2 trials (*p* = 0.004). Finally, we did not observe significant differences in terms of pHa and PaCO_2_ value in the different trials.

A tracheostomy was required in 4 patients (33%), who had a very long length of stay (LOS) in PICU (days: 80.5 ± 43.33), while the remaining 8 patients were successfully extubated and were discharged to the pediatric ward after a PICU-LOS of 45.5 ± 11.14 days. Two patients (16%) died from septic complications in PICU. After the end of the study, all patients were ventilated with NAVA until extubation or tracheostomy.

## Discussion

This physiological single center, unblinded, crossover study showed the clinical feasibility and the possible advantages in patient-ventilation synchrony and oxygenation of NAVA compared to PSV in a selected population of difficult to wean pediatric patients recovering from moderate PARDS. When compared to PSV, NAVA significantly reduced the AI, improved patient-ventilator synchrony and PaO_2_/FiO_2_ ratio, maintaining hemodynamic stability. To the best of our knowledge, this is the first clinical study exploring the effects of NAVA compared to PSV in a pediatric population with moderate PARDS presenting difficult weaning from mechanical ventilation.

Pediatric patients, especially after acute respiratory failure, present high respiratory rates, small tidal volumes, and strong inspiratory efforts. Patient-ventilator synchrony is difficult to achieve with PSV in this specific population [31]. Generally, pediatric patients spend about one-third of the respiratory time with a sub-optimal patient-ventilator interaction [32].

Several studies have reported that patients with PARDS are characterized by short ventilator free periods and longer length of mechanical ventilation in survivors [7]. Moreover, this specific population showed the worst patient-ventilator interaction [32–34].

As already demonstrated [32], the analysis of Edi signal facilitates the detection of patient-ventilator asynchrony, in particular for the calculation of timing errors for triggering or cycling-off.

As observed in adults [35, 36], all pediatric studies showed that patient-ventilator interaction unequivocally improved during both invasive and non-invasive NAVA compared to conventional modes such as PSV.

Also, during non-invasive ventilation (NIV), NAVA was compared to conventional NIV in PSV mode, showing a significant reduction of all major asynchronies [16].

Our results confirm that NAVA significantly reduces patient-ventilator asynchrony and significantly increases oxygenation compared to conventional assisted mechanical ventilation [37]. In fact, in our study, the better synchrony with NAVA was confirmed by a significantly longer Time_sync_ and substantially better Time_sync_/Ti_neu._ compared to PSV mode.

Other studies [14, 37] highlighted the positive effect of NAVA on PaO_2_/FiO_2_ ratio, but they did not show significant effects on PaCO_2_ as reported in our study, probably due to the recovery of a normalized respiratory pattern with normocapnia in patients recovering from PARDS.

Recent studies confirmed the clinical feasibility and advantages of NAVA in pediatric patients after cardiac surgery [38, 39] especially in patients with difficult weaning.

In the abovementioned study [39], as in other studies [14, 40], NAVA determined a significant reduction of peak inspiratory airway pressure and mean airway pressure when compared to conventional ventilation modes. In our study, peak inspiratory airway pressure was similar in all tested conditions, as we set the NAVA level to achieve similar peak inspiratory pressures to those observed during PSV.

The effect of NAVA on PeakEdi is particularly interesting: in a recent review, Karikari and colleagues [37] described how NAVA may affect the electrical activity of the diaphragm, although not significantly, maintaining the large variability of Edi, a characteristic observed in spontaneous breathing. In our study, we observed a trend toward a rise of peakEdi during NAVA. The peakEdi during PSV trials showed reduced values, as possible expression of over assistance in PSV mode. This ability of NAVA to maintain the characteristic variability of spontaneous breathing has been demonstrated in several studies [13, 37], that highlighted the significant effect of NAVA on tidal volume and airway pressure variability with respect to conventional ventilation. In our study, despite the observed improvement in patient-ventilator interaction with NAVA, this difference in tidal volume was not observed probably because the PSV setting used during our study, set to avoid a long Timech, allowed to deliver similar Vtmech and Vtneu in all conditions. For this reason, most of our patients presented a VTneu /VTmech of 100%.

We acknowledge that this study has several limitations. First, this was a single center study with a relatively small sample size, although in a highly selected population. Second, this study is not a controlled-randomized trial. Third, this physiological study is focused on the effects of NAVA in terms of patient-ventilator interaction and AI, without investigating the influence of NAVA on other variables such as patient comfort and sedation requirements. Further studies are required to better investigate the influence of these and other variables.

## Conclusions

In a specific pediatric ICU population presenting difficult weaning after PARDS, NAVA was associated with a reduction of the AI and an improvement of patient-ventilator interaction. Moreover, NAVA seems to be a safe alternative to PSV with the added advantage of improving oxygenation. The results of this physiological single center crossover study support the application of NAVA in patients showing difficult weaning when recovering from PARDS.

## Data Availability

The datasets used and/or analyzed during the current study are available from the corresponding author on reasonable request.

## Abbreviations

AC: Auto-cycling
AI: Asynchrony index
AT: Auto-trigger
ARF: Acute Respiratory Failure
bpm: beats per minute
CPAP: Continuous Positive Airway Pressure
Edi: Electrical activity of the diaphragm
FiO_2_: Fraction of the inspired oxygen
HFOV: High Frequency Oscillatory Ventilation
HR: Heart rate
Kg: Kilograms
LC: Late cycling
ml: milliliters
mmHg: millimetres of mercury
msec: milliseconds
min: minute
NAVA: Neurally Adjused Ventilatory Assist
NIV: Non-invasive Ventilation
OI: Oxygenation index
PaO2: Partial pressure of arterial oxygen
PaO_2_/FiO_2_ ratio: Partial pressure of arterial oxygen to fraction of the inspired oxygen ratio
PaCO_2_: Arterial partial pressure of carbon dioxide
PARDS: Pediatric Acute Respiratory Distress Syndrome
Paw: Airway Pressure
PBW: Predicted body weight
PCV: Pressure control ventilation
PEEP: Positive End Expiratory Pressure
pHa: Arterial pH
PICU: Pediatric Intensive Care Unit
PSV: Pressure Support Ventiation
PTPEdi: Pressure time product of Edi
RR: Respiratory rate
RR_mech_: Mechanical respiratory rate
Sec: Seconds
SBT: Spontaneous breathing trial
SpO_2_: Peripheral oxygen saturation
∫Edi: EAdi time integral
Ti_mech_: Mechanical inspiratory time
Ti_neu_: Neural inspiratory time
Te_mech_: Mechanical expiratory time
Te_neu_: Neural expiratory time
VAP: Ventilator Associating Pneumonia
Vt: Tidal Volume
Vt_mech_: Mechanical tidal volume
Vt_neu_: Neural tidal volume
V′: Airflow
WE: Wasted effort

## References

[1] Chiumello D, Pelosi P, Taccone P, Slutsky A, Gattinoni L (2003). Effect of different inspiratory rise time and cycling off criteria during pressure support ventilation in patients recovering from acute lung injury. Crit Care Med.

[2] Chiumello D, Polli F, Tallarini F, Chierichetti M, Motta G, Azzari S, Colombo R, Rech R, Pelosi P, Raimondi F, Gattinoni L (2007). Effect of different cycling-off criteria and positive end-expiratory pressure during pressure support ventilation in patients with chronic obstructive pulmonary disease. Crit Care Med.

[3] Gea J, Roca J, Torres A, Agustí AG, Wagner PD, Rodriguez-Roisin R (1991). Mechanisms of abnormal gas exchange in patients with pneumonia. Anesthesiology.

[4] Blanch L, Villagra A, Sales B, Montanya J, Lucangelo U, Lujan M, Garcia-Esquirol O, Chacon E, Estruga A, Oliva JC, Hernandez-Abadia A, Albaiceta GM, Fernandez-Mondejar E, Fernandez R, Lopez-Aguilar J, Villar J, Murias G, Kacmarek RM (2015). Asynchronies during mechanical ventilation are associated with mortality. Intensive Care Med.

[5] Thille AW, Rodriguez P, Cabello B, Lellouche F, Brochard L (2006). Patient-ventilator asynchrony during assisted mechanical ventilation. Intensive Care Med.

[6] De Haro C, Ochagavia A, López-Aguilar J, Fernandez-Gonzalo S, Navarra-Ventura G, Magrans R, Montanyà J, Blanch L (2019). Asynchronies in the Intensive Care Unit (ASYNICU) Group. Patient-ventilator asynchronies during mechanical ventilation: current knowledge and research priorities. Intensive Care Med Exp.

[7] Khemani RG, Smith L, Lopez-Fernandez YM, Kwok J, Morzov R, Klein MJ, Yehya N, Willson D, Kneyber MCJ, Lillie J, Fernandez A, Newth CJL, Jouvet P, Thomas NJ (2019). Pediatric acute respiratory distress syndrome incidence and epidemiology (PARDIE) investigators and the pediatric acute lung injury and Sepsis investigators (PALISI) network. Pediatric acute respiratory distress syndrome incidence and epidemiology (PARDIE): an international observational study. Lancet Respir Med.

[8] Sinderby C, Navalesi P, Beck J, Skrobik Y, Comtois N, Friberg S, Gottfried SB, Lindstrom L (1999). Neural control of mechanical ventilation in respiratory failure. Nat Med.

[9] Beck J, Sinderby C, Lindstro ML, Grassino A (1998). Effects of lung volume on diaphragm EMG signal strength during voluntary contractions. J Appl Physiol.

[10] Sinderby CA, Comtois AS, Thomson RG, Grassino AE (1996). Influence of the bipolar electrode transfer function on the electromyogram power spectrum. Muscle Nerve.

[11] Sinderby C, Lindstrom L, Comtois N, Grassino A (1996). Effects of diaphragm shortening on the mean action potential conduction velocity. J Physiol Lond.

[12] Lourenco RV, Cherniack NS, Malm JR, Fishman AP (1966). Nervous output from the respiratory centers during obstructed breathing. J Appl Physiol.

[13] Bordessoule A, Emeriaud G, Morneau S, Jouvet P, Beck J (2012). Neurally adjusted ventilatory assist improves patient–ventilator interaction in infants as compared with conventional ventilation. J Pediatr Res.

[14] Breatnach C, Conlon NP, Stack M, Healy M, O’Hare BP (2010). A prospective crossover comparison of neurally adjusted ventilatory assist and pressure-support ventilation in a pediatric and neonatal intensive care unit population. Pediatr Crit Care Med.

[15] Vignaux L, Grazioli S, Piquilloud L, Bochaton N, Karam O, Levy-Jamet Y, Jaecklin T, Tourneux P, Jolliet P, Rimensberger PC (2013). Patient-ventilator asynchrony during noninvasive pressure support ventilation and neurally adjusted ventilatory assist in infants and children. Pediatr Crit Care Med.

[16] Ducharme-Crevier L, Beck J, Essouri S, Jouvet P, Emeriud G (2015). Neurally adjusted ventilatory assist (NAVA) allows patient-ventilator synchrony during pediatric noninvasive ventilation: a crossover physiological study. Crit Care.

[17] Chidini G, De Luca D, Conti G, Pelosi P, Nava S, Calderini E (2016). Early noninvasive neurally adjusted ventilatory assist versus noninvasive flow-triggered pressure support ventilation in pediatric acute respiratory failure: a physiologic randomized controlled trial. Pediatr Crit Care Med.

[18] Pediatric Acute Lung Injury Consensus Conference Group (2015). Pediatric acute respiratory distress syndrome: consensus recommendations from the Pediatric Acute Lung Injury Consensus Conference. Pediatr Crit Care Med.

[19] Begg C, Cho M, Eastwood S, Horton R, Moher D, Olkin I, Pitkin R, Rennie D, Schulz KF, Simel D, Stroup DF (1996). Improving the quality of reporting of randomized controlled trials. The CONSORT statement. JAMA.

[20] Mathers LH, Frankel LR, Behrman RE, Kliegman RM, Jenson HB (2004). Stabilization of the critically ill child. Nelson Textbook of Pediatrics.

[21] Erwin I, Monique V, Dick T (2005). Assessment of sedation levels in pediatric intensive care patients can be improved by using the COMFORT “behavior” scale. Pediatr Crit Care Med.

[22] Boles JM, Bion J, Connors A, Herridge M, Marsh B, Melot C, Pearl R, Silverman H, Stanchina M, Vieillard-Baron A, Welte T (2007). Weaning from mechanical ventilation. Eur Respir J.

[23] Newth CJ, Venkataraman S, Willson DF, Meert KL, Harrison R, Dean JM, Pollack M, Zimmerman J, Anand KJ, Carcillo JA, Nicholson CE, Eunice Shriver Kennedy National Institute of Child Health and Human Development Collaborative Pediatric Critical Care Research Network (2009). Weaning and extubation readiness in pediatric patients. Pediatr Crit Care Med.

[24] Colombo D, Cammarota G, Bergamaschi V, De Lucia M, Corte FD, Navalesi P (2008). Physiologic response to varying levels of pressure support and neutrally adjusted ventilatory assist in patients with acute respiratory failure. Intensive Care Med.

[25] Sinderby CA, Beck JC, Lindström LH, Grassino AE (1997). Enhancement of signal quality in esophageal recordings of diaphragm EMG. J Appl Physiol.

[26] Cammarota G, Longhini F, Perucca R, Ronco C, Colombo D, Messina A, Vaschetto R, Navalesi P (2016). New setting of neurally adjusted ventilatory assist during noninvasive ventilation through a helmet. Anesthesiology.

[27] Olivieri C, Longhini F, Cena T, Cammarota G, Vaschetto R, Messina A, Berni P, Magnani C, Della Corte F, Navalesi P (2016). New versus conventional helmet for delivering noninvasive ventilation: a physiologic, crossover randomized study in critically ill patients. Anesthesiology.

[28] Longhini F, Pan C, Xie J, Cammarota G, Bruni A, Garofalo E, Yang Y, Navalesi P, Qiu H (2017). New setting of neurally adjusted ventilatory assist for noninvasive ventilation by facial mask: a physiologic study. Crit Care.

[29] Passath C, Takala J, Tuchscherer D, Jakob SM, Sinderby C, Brander L (2010). Physiologic response to changing positive end-expiratory pressure during neurally adjusted ventilatory assist in sedated, critically ill adults. Chest.

[30] de la Oliva P, Schüffelmann C, Gómez-Zamora A, Villar J, Kacmarek RM (2012). Asynchrony, neural drive, ventilatory variability and COMFORT: NAVA versus pressure support in pediatric patients. A non-randomized cross-over trial. Intensive Care Med.

[31] Beck J, Reilly M, Grasselli G, Mirabella L, Slutsky AS, Dunn MS, Sinderby C (2009). Patient–ventilator interaction during neurally adjusted ventilatory assist in low birth weight infants. Pediatr Res.

[32] Mortamet G, Larouche A, Ducharme-Crevier L, Fléchelles O, Constantin G, Essouri S, Pellerin-Leblanc AA, Beck J, Sinderby C, Jouvet P, Emeriaud G (2017). Patient-ventilator asynchrony during conventional mechanical ventilation in children. Ann Intensive Care.

[33] Blokpoel RG, Burgerhof JG, Markhorst DG, Kneyber MC (2016). Patient–ventilator asynchrony during assisted ventilation in children. Pediatr Crit Care Med.

[34] Colombo D, Cammarota G, Alemani M, Carenzo L, Barra FL, Vaschetto R, Slutsky AS, Della Corte F, Navalesi P (2011). Efficacy of ventilator waveforms observation in detecting patient– ventilator asynchrony. Crit Care Med.

[35] Demoule A, Clavel M, Rolland-Debord C, Perbet S, Terzi N, Kouatchet A, Wallet F, Roze H, Vargas F, Guerin C, Dellamonica J, Jaber S, Brochard L, Similowski T (2016). Neurally adjusted ventilatory assist as an alternative to pressure support ventilation in adults: a French multicentre randomized trial. Intensive Care Med.

[36] Ferreira JC, Diniz-Silva F, Moriya HT, Alencar AM, Amato MBP, Carvalho CRR (2017). Neurally Adjusted Ventilatory Assist (NAVA) or Pressure Support Ventilation (PSV) during spontaneous breathing trials in critically ill patients: a crossover trial. BMC Pulm Med.

[37] Karikari S, Rausa J, Flores S, Loomba RS (2019). Neurally adjusted ventilatory assist versus conventional ventilation in the pediatric population: Are there benefits?. Pediatr Pulmonol.

[38] Crulli B, Khebir M, Toledano B, Vobecky S, Poirier N, Emeriaud G (2018). Neurally adjusted ventilatory assist after pediatric cardiac surgery: clinical experience and impact on ventilation pressures. Respir Care.

[39] Bonacina D, Bronco A, Nacoti M, Ferrari F, Fazzi F, Bonanomi E, Bellani G (2019). Pressure support ventilation, sigh adjunct to pressure support ventilation, and neurally adjusted ventilatory assist in infants after cardiac surgery: A physiologic crossover randomized study. Pediatr Pulmonol.

[40] Liet J-M, Barrière F, Roux BG-L, Bourgoin P, Legrand A, Joram N (2016). Physiological effects of invasive ventilation with neurally adjusted ventilatory assist (NAVA) in a crossover study. BMC Pediatr.

